# Radiation Therapy for the Treatment of Recurrent Glioblastoma: An Overview

**DOI:** 10.3390/cancers4010257

**Published:** 2012-03-07

**Authors:** Dante Amelio, Maurizio Amichetti

**Affiliations:** ATreP—Agenzia Provinciale per la Protonterapia, Via F.lli Perini 181, Trento 38122, Italy; E-Mail: amichett@atrep.it

**Keywords:** glioblastoma, recurrence, radiation therapy

## Abstract

Despite the therapeutic advances in neuro-oncology, most patients with glioblastoma ultimately experience local progression/relapse. Re-irradiation has been poorly viewed in the past, mainly due to the overestimated risk of side effects using conventional radiotherapy. To date, thanks to the improvement of several delivery techniques, together with improved imaging capabilities, re-irradiation is a viable salvage treatment option to manage such clinical scenario. A literature overview on the feasibility and efficacy of the different irradiation modalities for recurrent glioblastoma along with considerations on areas of improvement are provided.

## 1. Introduction

Glioblastoma (GBM) is the most frequent primary malignant brain tumor in adults [1]. Multimodality treatment with surgical resection followed by adjuvant radiation therapy (RT) and chemotherapy (CHT) represents the current standard of care [2]. The recently updated EORTC/NCIC randomized trial [3] has shown unequivocally that addition of temozolomide (TMZ) to RT provides both progression-free and overall survival advantage with respect to RT alone. Nevertheless, the prognosis is still dismal being the median and 2-year overall survival of the combined modality 14.6 months and 27%, respectively [3]. Despite of this aggressive multimodality strategy long-term control of such malignancy is rarely achieved and it ultimately recurs within 2 cm of the resection margin in nearly all patients [4].

Limited approaches are currently available for the salvage treatment of GBM patients recurring after primary treatment, including surgical re-resection [5,6,7,8,9], chemotherapy [10,11] or re-irradiation. Moreover, at the time of recurrence the location and size of the tumor as well as the patient clinical status hamper taking advantage from either modality and there is no standard of care yet.

Nowadays, patients with recurrent GBM have almost certainly received a full course of RT during the primary treatment. Until recently, the risk of severe re-irradiation morbidity has limited the employment of a second irradiation. Palliative re-irradiation to moderate doses might be feasible without using very advanced techniques. However, under many circumstances two-dimensional or three-dimensional conformal therapy do not fulfill the required normal tissues constraints. The improvement of imaging modalities [12] and the development of high-precision RT techniques [13] have allowed better target definition and more accurate radiation delivery. This ultimately enabled the safe administration of a second course of irradiation. The radiation tolerance of normal tissue is reduced compared with the first radiotherapy course unless complete repair of radiation damage has occurred. *In vivo* radiobiological data suggest that after an initial course of RT, brain tissue may repair the radiation-related damage depending on the primary total dose and fractionation as well as the time lapse between treatments [14]. Numerous cellular enzymatic mechanisms can directly repair damaged DNA, or allow tolerance of DNA lesions ultimately reducing potential harmful effects. Unfortunately, the exact mechanism underlying such recovery is not clearly understood yet. The recovery capacity is the main determinant of the size of the re-irradiation dose depending on the initial biologically effective dose (BED). Because of the low repair capacity of the normal brain (reflected by the so called α/β ratio, which is estimated to be approximately 2 Gy), the BED rather than the physical irradiation dose should be considered in re-irradiation protocols. Such a possibility could further reduce the risk of severe side effects consequent to re-irradiation. The cumulative tolerance dose of normal brain tissue delivered in 2 Gy per fraction (EQD2_cumulative_) approximates 100 Gy [15]. Moreover, the applied re-irradiation dose and EQD2_cumulative_ were found to increase with a change in irradiation technique from conventional to more conformal techniques (like fractionated stereotactic radiotherapy and radiosurgery) without increasing the probability of normal brain necrosis [15]. So far, radiation oncologists can exploit many techniques such as three-dimensional conformal RT (3D-CRT), fractionated stereotactic RT (FSRT), stereotactic radiosurgery (SRS), brachytherapy (BT), intensity-modulated RT (IMRT), and particle therapy (PT), which may be helpful to face GBM patient re-irradiation.

Aim of the present article is to provide an overview on the different techniques for re-irradiation of recurrent GBM, while highlighting the technical and clinical rationale for application as well as the corresponding clinical outcomes. Further considerations on potential study weaknesses and areas of improvement are also provided.

## 2. Results

### 2.1. Study Selection and Inclusion Criteria

In order to provide a comprehensive review of the published literature regarding re-irradiation of GBM the PubMed and MEDLINE databases were searched. Articles were retrieved using the following keywords: “glioblastoma”, “recurrent”, “radiotherapy”, “intensity-modulated radiation therapy”, “fractionated stereotactic radiotherapy”, “radiosurgery”, “brachytherapy”, “gliasite”, “particle therapy”, “radioimmunotherapy”, and “boron neutron capture therapy”. Only studies published from the beginning of 1990 through the end of June 2011 and providing clinical results of ten or more recurrent GBM patients were included. The search was limited to articles in English language. Review articles, editorials, case reports, letters of opinion, and congress abstracts were excluded, even if they added valuable information. In case of repeated publications from the same institution, only the most updated was used for the analysis. Multiple publications from the same institution were included if reporting patients treated over different time periods. Considering that recurrent GBM (World Health Organization grade 4) are usually pooled and analyzed together with recurrent anaplastic gliomas (World Health Organization grade 3), and that tumor grade may represent a relevant prognostic factor [16], only studies distinguishing clinical outcomes according to the tumor histology were included.

A systematic review was beyond the aim of the paper. In the following results are reported in the form of a narrative synthesis.

### 2.2. Conventional External Beam Radiation Therapy

The potential of 3D-CRT for re-irradiation of selected intracranial tumors was evaluated in the clinical practice at the beginning of the Nineties. In fact, the development of the 3D technology allowed the practical integration of computed tomography (CT) and/or magnetic resonance (MR) imaging into treatment planning and the development of personalized blocks shielding the healthy tissues while conforming to the tumor. Moreover, it is an outpatient-based, non-invasive and non-complex technique that takes advantage of the properties of a standard fractionation schedule. In fact, the dose fractionation allows for the re-oxygenation of the tumor tissue [17] as well as the re-distribution of tumor cells into sensitive cell cycle phases [18]. Finally, because of different tumor radiobiological behaviour with respect to surrounding nervous tissues fractionation provides effective tumor killing while reducing the risk of healthy tissues late side effects [19]. With this 3D planning process, conformal external beam RT was applied more frequently to the re-irradiation of patients with recurrent gliomas. Nevertheless, the employment of only few beams and a sub-optimal radiation collimation does not best spare neighbouring tissues and ultimately allows the delivery of relatively low dose in this clinical scenario.

Despite several series on re-irradiation of high-grade gliomas with 3D-CRT have been published very few studies focused or reported specific outcomes concerning GBM re-irradiation by this technique [20,21,22] for a total of 67 patients. There were no prospective trials.

Several fractionation schemes were registered: Veninga *et al*. delivered a median dose of 46 Gy by conventional fractionation [21], Nieder *et al*. treated the patients bis-in-die up to 45.5 Gy [20], and Henke *et al*. employed hypofractionated RT to deliver a median dose of 20 Gy [22]. In one study only [22], some chemotherapeutic regimens were combined with irradiation.

Overall, the treatment was quite well tolerated and only a limited radionecrosis rate (2–6%) was reported even though data refer both to GBM and anaplastic glioma patients. The reported median overall survival (OS) was in the range of 6–10 months.

The few published data concerning patients re-irradiated by this technique demonstrated the feasibility of a second treatment performed on limited fields and pointed out acceptable side effect rates, whereas the clinical outcomes were quite satisfying. To date, this technique should be employed to deliver short-course palliative re-irradiation in patients with worse prognostic factors.

### 2.3. Fractionated Stereotactic Radiation Therapy

The principles of stereotactic RT were developed in the fifties by Leksell [23]. At the beginning, the dose was applied only in a single fraction (SRS). In the eighties, the development of re-locatable frames and specific delivery systems allowed also linear accelerator-based stereotactic treatments. In fact, the employment of circular and micro-multileaf collimators provides a better target dose conformity together with a steep dose gradient between the tumor and surrounding normal tissues, which reduces the risk of radiation-related side effects and ultimately improves the therapeutic ratio. Moreover, thanks to the high-level accuracy and reproducibility, it is still possible to exploit the radiobiological advantages of fractionation (FSRT). FSRT can be delivered with standard fractionation regimens or with hypofractionated schedules. Hence, also larger tumors, which might be technically ineligible for other techniques (implantation or SRS), can be safely and effectively treated.

Finally, it is noteworthy that FSRT can be delivered as an outpatient-based, non-invasive approach. Such a possibility is not only more beneficial to patients with respect to quality of life and convenience, but it may also represent a decrease in costs associated with retreatment.

Fifteen reports [24,25,26,27,28,29,30,31,32,33,34,35,36,37,38] are available in the literature that focused or reported specific outcomes concerning GBM re-irradiation by FSRT (data summarized in Table 1).

All but two [29,34] were retrospective. Median age was between 39 and 61 years with median Karnofsky performance status (KPS) ranging between 70 and 90, even though also patients with lower KPS (40–50%) were treated in almost all reports. The median target volume presented a wide range encompassed between 5.7 and 51.1 cc. However, also very large relapses (>100 cc) were treated. Dose of re-irradiation varied between hypofractionated schedules with single doses >4 Gy [24,26,28,29,32,34], moderately hypofractionated schemes with the use of 3–3.5 Gy per fraction [25,32,36] or conventionally fractionated doses [27,35,37,38]. Median total doses delivered ranged widely between 20 and 37.5 Gy.

Overall, most series pointed out an OS of 8–12 months. Seven papers [26,27,28,29,31,34,37] provided data concerning progression-free survival (PFS). In most series, the median value ranged between 3 and 5.6 months. Only Gutin *et al*. achieved a median PFS of 7.3 months [34].

In six studies [24,29,31,34,36,37], different types of chemotherapy (TMZ, topotecan, taxol, bevacizumab) were combined with radiotherapy. Median OS was similar in patients treated with radiotherapy alone (range, 7–13.4 months; median value, 9.7 months) and with concomitant chemotherapy (range, 4–11 months; median value, 9 months).

The analysis of data regarding the detection of prognostic factors pointed out that, at the multivariate analysis, GTV < 20 cc and dose > 30 Gy [25], surgical intervention before re-irradiation [31], time to re-irradiation and extent of second surgery [35], younger age and smaller GTV [36] as well as O^6^-methylguanine-DNA-methyltransferase methylation status [37] predicted for better OS.

**Table 1:** Re-irradiation series employing fractionated stereotactic radiation therapy.

Author	≠ GBM Pts	Med Age in Years	Med KPS (range) in %	Surg before Re-Irr in %	Med TD before Re-Irr in Gy	Med Time to Re-Irr in Months	Med re-irr TD (range)/dpfx in Gy	Med Vol (range) in cc	CHT	Med Survival from Re-Irr in Months	Side Effects
Lederman *et al*. [24]	14	* 56	* 70 (50–100)	NR	* 60	* 7.8	* 24/6	* 32.7 (1.5–150)	TAX	OS 7	* RN 8% * Reop 13%
Hudes *et al*. [25]	19	* 52	* 80 (60–100)	NR	* 60	* 3.1	range 24–35/3–3.5	* 12.6 (0.89–47.5)	--	OS 10.5	* steroid increase 15%
Selch *et al*. [26]	14	61	70 (50–90)	STR 21	60	11	25 (20–45)/4–6	11.6 (9–17)	--	OS 4 PFS 4	No
Combs *et al*. [27]	53	55	≥80: 46	NR	57	10	36/2	49 (7.5–632) (PTV)	--	OS 8 PFS 5	No > G2
Vordemark *et al*. [28]	14	* 50	* 90 (60–90)	* 63 (NS)	* 45–61	* 19	*30 (20–30)/4–10	* 15 (4–70)	--	OS 7.3 PFS 4.6	* reop 5%
Wurm *et al*. [29]	20	* 45	*80 (50–100)	NR	* 54.4 bid/60	* 12.8	*range 25–30/5–6	* 16.5 (1–70.9)	Topo	OS 7.9 PFS 5.6	* G2 RTOG 12%
Kohshi *et al*. [30]	11	* 46	*70 (40–100)	NR	* 60	* 11	*22 (18–27)/2.25–3.3	* 8.7 (1.7–159)	--	OS 11	Reop 18%
Combs *et al*. [31]	25	39	≥70: 92%	GTR 20 STR 52	60	36	36 (25–45)/2	50 (16–49)	TMZ	OS 8 PFS 5	No
Fokas *et al*. [32]	53	53	70	43 (NS)	54	NR	30 (20–60)/2–5	35 (3–204)	--	OS 9	No
Patel *et al*. [33]	10	44	90 (70–90)	GTR 20 STR 40	50–60	14.9	36/6	51.1 (16.1–123.3)	--	OS 7.4	RN 10% Reop 10%
Gutin *et al*. [34]	2	56	*80 (70–100)	NR	* 59.4	* 15	*30/6	* 34 (2–62)	Beva	OS 12.5 PFS 7.3	* Reop 12% * hemorrhage 4% * wound dehiscence 4%
Villaceincio *et al*. [35]	26	56	80 (70–100)	GTR 57 STR 34	59.4	13	§ 20 (8–25)	7 (0.4–48.5)	--	OS 7	NR
Fogh *et al*. [36]	105	NR	NR	* GTR 16 * STR 41	60	8	*35/3.5	* 22 (0.6–104)	* 48 various	OS 11	* steroid increase 10%
Minniti *et al*. [37]	36	56	70 (60–100)	NR	60	14	37.5/2.5	13.1 (1–35.3)	TMZ	OS 9.7 PFS 3	RN 8%
Maier-Hauff *et al*. [38]	59	55.7	90 (60–100)	18 (NS)	NR	NR	30/2 + HT	46.5 (6.6–108)	--	OS 13.4	No

Med: median; pts: patients; TD: total dose; GBM: glioblastoma; re-irr: re-irradiation; dpfx: dose per fraction; NR: not reported; NS: not specified; vol: volume; GTR: gross total resection; STR: subtotal resection; RN: radionecrosis; Gy: Gray; OS: overall survival; PFS: progression-free survival; CHT: chemotherapy; KPS: Karnofsky performance status; RN: radionecrosis; reop: reoperation; TMZ: temozolomide; Beva: bevacizumab; Topo: topotecan; TAX: paclitaxel; HT: thermotherapy; G: grade; RTOG: Radiation Therapy Oncology Group; PTV: planning target volume; *: data refer to all patients analyzed and include high-grade gliomas; § delivered in 1 to 5 fractions (median 2).

Data regarding toxicity were available in 14 studies. Only three studies reported the occurrence of radionecrosis and five of reoperation. Unfortunately, these data often refer to mixed samples including both GBM and non-GBM patients.

Based on these data, FSRT appears a feasible and safe re-irradiation technique even when the target has sizeable volume. Despite target volumes were generally larger than those reported in SRS/BT series patients treated with FSRT had comparable survival. At the light of this remark, FSRT may be a better option for patients with large tumors or tumors in eloquent structures.

### 2.4. Stereotactic Radiosurgery

Stereotactic radiosurgery is a non-invasive irradiation modality that can be delivered with Gamma Knife (Elekta, Stockholm, Sweden), Cyberknife (Accuracy, Sunnyvale, CA, USA), or specially adapted linear accelerators without relevant dosimetrical differences [39]. It is a highly conformal, precise and accurate technique. Hence, the main advantage of SRS is the capability of relevant dose delivery to the tumor volume while sparing surrounding normal tissues. From the radiobiological standpoint, SRS exploits a different pattern of dose distribution rather than the radiobiological differences between normal and tumor tissue. In fact, the argument for the use of SRS is the relevant radiobiological effect of single-session radiation cell kill or cell division capability arrest, regardless of the mitotic phase. Moreover, it has been argued that when the treatment volume is small and contains little functioning brain tissue, the need for fractionation may not apply [40]. Considering that treatment-related toxicity increases with target size as well as increased delivered dose, the lesions amenable by SRS are usually small and not at close proximity to eloquent structures (e.g., optic pathway, basal ganglia, speech or motor area). However, also deep-sited lesions (usually considered not implantable) can be managed.

Radiosurgery is an outpatient-based technique that reduces treatment and hospitalization times. The application of radiation takes place without surgical procedures. As a consequence, many of the risks involved with brachytherapy (such as infection, hemorrhage, exposure of the personnel to radiation) do not apply to SRS. More recently, the development of image-guided RT and frameless SRS systems has provided good positioning accuracy and clinical efficacy demonstrating the possibility for a further improvement of patient compliance [41,42].

Between 1992 and 2011 several papers [33,43,44,45,46,47,48,49,50,51,52,53] pointed out the results regarding SRS re-irradiation of recurrent GBM (data summarized in Table 2).

All but four [45,47,51,53] were retrospective. In general, suitable patients were fairly young, with a high KPS and small relapses. However, it is noteworthy that SRS was exploited also in patients with poor clinical status (KPS 40–50%) and large recurrent tumors (60–80 cc). As a consequence, even though the median prescribed dose had very limited variations (13–18 Gy) the delivered dose range was much larger (5–50 Gy).

**Table 2:** Re-irradiation series employing stereotactic radiosurgery.

Author	≠ GBM Pts	Med Age in Years	Med KPS (range) in %	Surg before Re-Irr in %	Med TD before Re-Irr in Gy	Med Re-Irr TD (range) in Gy	Med Interval to Re-Irr in Months	Med Vol (range) in cc	Med Survival from Re-Irr in Months	Side effects
Shrieve *et al*. [43]	86	46	80 (40–100)	NR	NR	13 (6–20) to med 80% isodose (Linac)	10.3	10.1 (2.2–83)	OS 10.2	Seizures 3.5% hosp. 2.5% exitus 1% cr. nerve deficit 1% reop 22% RN 0%
Larson *et al*. [44]	46	53	§ 90 (40–100)	NR	NR	Med min 16 (5–37.5) to med 50% isodose (GK)	>16 weeks	§ 6.2 (0.3–96)	OS 57 weeks	NR
Kondziolka *et al*. [45]	19	§ Mean 51	§ Mean 90 (50–100)	NR	Mean 60	§ Mean 15.5 (12–25) to 50% isodose (GK)	18.9	§ Mean 6.5 mL (0.88–31.2)	OS 30	§ Reop 19% § RN 2%
Park *et al*. [46]	23	53	80	NR	NR	15 to 60% isodose (Linac/GK)	NR	9.9	OS 10.3 PFS 4.7	NR
Larson *et al*. [47]	14	53	90 (70–100)	NS	NR	Med min 15 (12–17.5) (GK)	12	8 (1.6–29.7)	OS 38 weeks PFS 15 weeks	
Combs *et al*. [48]	32	56	80 (70–100)	NR	54	15 (10–20) to 80% isodose (Linac)	10	10 mL (1.2–59.2)	OS 10 PFS 5	No > CTC G2 (Acute) RN 0%
Hsieh *et al*. [49]	26	58	§ Mean 70 (60–100)	NR	60	12 to 50% isodose (GK)	NR	Mean 21.6	OS 10	§ RN 31.3%
Mahajan *et al*. [50]	41	54	80 (70–100)	53.6 (NS)	60	NR (Linac)	10	4.7 (0.15–16.3)	OS 11	Reop 22% RN 2.4%
Kong *et al*. [51]	65	* 49	* 80 (50–100)	NR	60	* 16 (12–50) to 50% (GK) or 80% (linac) isodose (Linac/GK)	NR	* 10.6 mL (0.09–79.6)	OS 13 PFS 4.6	Reop 3.5% RN 24% (imaging-based)
Biswas *et al*. [52]	18	§ 57.8	≥ 70	NR	60	15 (9–20) to the isocenter (Linac)	12.1	8.4 mL (0.2–32)	OS 5.3 PFS 3.4	No > RTOG G2 (Acute)
Patel *et al*. [33]	26	53	80 (50–100)	GTR 4STR 38	Range 50–60	18 (12–20) to 90% isodose (Linac)	12.5	10.4 (0.3–60.1)	OS 8.4	NS
Maranzano *et al*. [53]	13	55 @	90 (70–100) @	NR	60	17 (14–22) to the isocenter (Linac)	9	5.3 (0.6–14)	OS 11	No > G2 (Acute) RN 23%

Med: median; pts: patients; GBM: glioblastoma; surg: surgery; TD: total dose; re-irr: re-irradiation; min: minimum; vol: volume; GK: gamma-knife; Linac: linear accelerator; NR: not reported; NS: not specified; OS: overall survival; PFS: progression-free survival; reop: reoperation; GTR: gross tumor resection; STR: subtotal resection; hosp: hospitalization; cr.: cranial; RN: radionecrosis; Gy: Gray; KPS: Karnofsky performance status; RTOG: radiation therapy oncology group; CTC: common toxicity criteria; G: grade; §: data refer to all patients analyzed and include both newly and recurrent high-grade gliomas; *: data refer to all patients analyzed and include high-grade gliomas; @: data refer to patients treated both with SRS and FSRT.

Overall, the use of SRS translated into reported median OS from re-irradiation of 10–13 months. Kondziolka *et al*. [45] and Biswas *et al*. [52] reported much better (30 months) and much worse (5.3 months) results, respectively. Apparently, there are no reasons that could justify such a difference. Only five papers [46,47,48,51,52] provided data concerning PFS with median values ranging between 3.4 and 5 months. Considering the above-mentioned patient homogeneity, it is not surprising that almost all the series provided very consistent outcomes. Accordingly, such homogeneity together with the lack of histologically-based subgroup analysis hampered the detection of well-defined prognostic factors. Concerning OS, only one study [43] pointed out the prognostic value of younger age (<46 years) as well as tumor volume (<10.1 cc) at multivariate analysis.

The treatment-related neurological side effects were generally mild. Nevertheless, almost all series pointed out the reoperation rates of 14–22%. The corresponding radionecrosis rates usually ranged between 2 and 5%. However, three series [49,51,53] pointed out values up to 31%. Considering the treatment was always delivered in a single fraction, there was no concomitant chemotherapy administration.

Based on the reported data, SRS is a feasible and effective irradiation technique in this clinical scenario. However, the risk of radionecrosis should not be underestimated; hence the patients should be carefully selected reserving SRS for small lesions. Unfortunately, a clear volume-cutoff cannot be defined from literature data so far.

### 2.5. Brachytherapy

Interstitial BT employing radioactive sources has been performed in recurrent GBM because its high spatial dose localization can improve the therapeutic ratio. In fact, as SRS and FSRT, BT allows the delivery of a large dose to the tumor volume while sparing surrounding normal tissue. Usually, it is delivered following the resection of recurrent tumors. However, the placement of multiple sources in the proximity of a resection cavity or relapsed tumor is challenging, and optimal dose distribution may be consequently difficult to be achieved [54]. Several sources such as 125-I, 192-Ir and 198-Au were employed to deliver high-dose (HDR) or low-dose-rate (LDR) irradiation as well as permanent or temporary implants. Theoretically, such techniques might differ in terms of toxicity risk. In fact, the use of low-dose-rate interstitial BT could reduce the rate of severe complications in comparison with high-dose-rate implants. From this standpoint, a novel alternative temporary BT system (Gliasite, Cytic Surgical Products, Palo Alto, CA, USA), which works as a single spherical source of low-dose-rate radiation, could overcome the limiting factors of conventional interstitial BT. In fact, the inflatable balloon can best fit with the resection cavity allowing the homogenous delivery of a steep dose gradient around the tumor bed. Considering that the radiation dose is usually delivered during four to six days, the radiobiological advantages of BT include re-oxygenation and accumulation of tumor cells into sensitive phases of the cell cycle.

Finally, it is to note that the invasive procedures dealing with BT involve some surgical risks (such as infection, hemorrhage as well as exposure of the personnel to radiation) and require the patient’s hospitalization.

Albeit the technical complexity in performing brachytherapy implants had hampered its use in the clinical practice, there is a wealth of experience on this topic [43,55,56,57,58,59,60,61,62,63,64,65,66] probably in relation to the above-mentioned intra-modality variability (data summarized in Table 3).

**Table 3:** Re-irradiation series employing brachytherapy.

Author	≠ GBM Pts	Med Age in Years	Med KPS (range) in %	Surg before Re-Irr in %	Med TD before Re-Irr in Gy	Med Re-Irr TD (range) in Gy	Med Interval to Re-Irr in Months	Med vol (range) in cc	Med survival from re-irr in months	Side effects
Scharfen *et al*. [55]	65	§ Mean 46	§ 90 (70–100)	NR	§ 60	§ 64.4 (37–120) Temp LDR 125-I sources	NR	NR	OS 49 weeks	§ G3 6% § G4 1% § G5 < 1% (Acute) § RN 5% § Reop 38%
Shrieve *et al*. [43]	32	45	80 (50–100)	NR	NR	50 (38.7–63.6) Temp LDR 125-I sources	7.3	29 (5–83)	OS 11.5	Scalp infections 6% (Acute) visual deficit 6% reop 44% RN 6%
Simon *et al*. [56]	42	49	80 (50–100)	B 100	Range 46–60	50 (15–60) Temp LDR 192-Ir sources	NR	23 (1.6–122)	OS 50 weeks	Skin necrosis 4.7% meningitis 9.5% reop 24% RN 7%
Tselis *et al*. [57]	84	57	80 (50–100)	NR	Up to 60	40 (30–50) Temp HDR 192-Ir sources	NR	51 (3–207)	OS 37 weeks	Intracerebral bleeding 2.3% meningitis 1.1% (Acute) RN 2.3%
Larson *et al*. [58]	13	55	NR	Max safe res 100	NR	Range 40–50 Perm LDR 198-Au seeds	NR	NR	OS 9	Reop 0% RN 0%
Halligan *et al*. [59]	18	41	90 (50–100)	GTR 83 STR 17	Range 54–64.8	210 (150–300) Perm LDR 125-I seeds	47 weeks	NR	OS 64 weeks	Reop 0% RN 0%
Gaspar *et al*. [60]	37	* 47	* 80 (60–100)	Max safe res 92 B 8	Range 50–66	103.68 Perm LDR 125-I seeds	NR	* 17 (3.9–78.8)	OS 10.8	* Reop 40% * RN 5%
Patel *et al*. [61]	40	50	70 (40–100)	GTR 55 STR 45	60 (all pts)	Range 120–160 Perm LDR 125-I seeds	NR	47.3 (7.5–91.1)	OS 47 weeks PFS 25 weeks	Healing complications 5% Reop 0% RN 0%
Larson *et al*. [62]	38	47	90 (60–100)	STR 60 (residual ≥ 0.5 cm) STR 40 (residual < 0.5 cm)	60	300 (150–500) Perm LDR 125-I seeds	39 weeks	21 (1–68, pre-implant)	OS 52 weeks PFS 16 weeks	Reop 10% RN 3%
Darakchiev *et al*. [63]	34	53	80 (60–90)	GTR 85 STR 15	NS	@ 120 Perm LDR 125-I seeds	NR	34(8–90, before surgery)	OS 69 weeks PFS 47 weeks	Healing complications 11.7% Reop 29% RN 23%
Tatter *et al*. [64]	15	Mean * 48.4	* 80 (60–100)	Max safe res 100	NS	Range 40–60 GliaSite-Iotrex	NR	NR	OS 8	Pseudomeningocele 4.7% wound infection 4.7% chemical meningitis 4.7%
Chan *et al*. [65]	24	48	80 (60–100)	Max safe res 100	Mean 59.8	Mean 53.1 (29.9–80) GliaSite-Iotrex	NR	≤30 (selection criteria)	OS 9.1	G1–2 headache 42% Nausea-vomiting 4% wound infections 6% (Acute) Neurological deficit 4% RN 8%
Gabayan *et al*. [66]	80	52	80 (40–100)	Max safe res 100	60	60 (38–72.5) GliaSite-Iotrex	* 40.6 weeks	< 5 cm diam (selection criteria)	OS 35.9 weeks * PFS 18.7 weeks	* G1 1.1% * G2 8.4% * G3 2.1% (RN)

Legend. Med: median; pts: patients; GBM: glioblastoma; surg.: surgery; TD: total dose; re-irr: re-irradiation; vol: volume; NR: not reported; NS: not specified; OS: overall survival; PFS: progression-free survival; reop: reoperation; RN: radionecrosis; Gy: Gray; KPS: Karnofsky performance status; diam: diameter; max: maximal; res: resection; GTR: gross total resection; STR: sub-total resection; B: biopsy; perm: permanent; temp: temporary; LDR: low-dose rate; HDR: high-dose rate; pts: patients; I: iodium; Ir: iridium; Au: gold; §: data refer to all patients analyzed and include both newly and recurrent high-grade gliomas; *: data refer to all patients analyzed and include high-grade gliomas; @: delivered in combination with BCNU wafers.

Unfortunately, all but two [63,64] were retrospective studies. Similarly to SRS, patients offered BT represented a selected population due to their favourable features. In general, they were young and in good clinical condition. Nevertheless, BT was employed also in patients with a KPS of 40–50%. Median tumor volume was usually limited in size even though also large relapses (80–90 cc) were implanted. It is noteworthy that treated volumes were generally larger than those reported in SRS series. Concerning the prescribed dose the use of either permanent or temporary implants and the delivery of both LDR and HDR irradiation ultimately generated a great variability. Most series employing temporary implants pointed out a median dose of 50–60 Gy regardless the dose-rate. However, depending on the tumor volume, the delivered dose ranged between 15 and 63.6 Gy. Only one series reported a maximum value of 120 Gy [55]. Conversely, in most series harnessing permanent implants (always LDR) the median dose was 120–130 Gy with maximum values up to 500 Gy.

Overall, the studies provided a median OS from re-irradiation of 10–12 months. Halligan *et al*. [59] and Darakchiev *et al*. [63] reported values of 14.9 and 16.1 months, respectively. In both series, a very high rate of gross tumor resection (>80%) before implantation may justify these favourable results. Only four articles [61,62,63,66] reported data concerning PFS with most median values ranging between 3.7 and 5.8 months. Darakchiev *et al*. pointed out a median value of 10.9 months [63]. Again, the good quality of pre-implantation surgical excision could explain such results. It is of note that outcomes are very consistent regardless the dose-rate, isotope, and implant modality. Moreover, most series detected some prognostic factors. At the multivariate analysis re-operation after re-irradiation [55], younger age [60,62,63], KPS ≥ 70 [63,65,66], and tumor volume < 17 cc [60] predicted better OS while younger age [61], KPS ≥ 70 [62,63], and gross tumor resection before implantation [61] predicted better local control.

In general, such results were achieved at the expense of mild neurologic toxicity. Nevertheless, most series pointed out relevant reoperation rates (10–40%). Radionecrosis rate was reported in 12 studies. In three series [58,59,61] the pathological analysis did not find any; Darakchiev *et al*. reported a radionecrosis rate of 23% [63]; radionecrosis rate was between 2 and 8% in the remaining studies. The level of radionecrosis was observed independently by dose-rate, isotope, and implant modality used. Specific modality-related side effects such as wound infections, skin necrosis, healing complications, meningitis, and cerebrospinal fluid leak were recorded up to 10% of cases.

Local chemotherapy was administered as part of a re-treatment strategy only in one report [63]. The role of chemotherapy administration was not addressed.

Based on these data, BT provides encouraging results even though they have to be interpreted in the light of the relevant reoperation rate before implantation. Moreover, the procedures involve some surgical risks. The relevant intra-modality variability hampered the possibility to address all the issues dealing with this technique so that the optimum prescribed dose, dose-rate, isotope, and implant modality have yet to be properly clarified. Better results can be expected in younger patients with a good functional status and small no-deep lesions, which may represent the best application setting. Implantation of large tumors (even though feasible) should be avoided.

### 2.6. Other Techniques

Apart from the above-mentioned and widely used techniques, further irradiation modalities such as radioimmunotherapy (RIT) and boron neutron capture therapy (BNCT) have been tested in recurrent GBM in prospective phase I-II trials providing preliminary results. There were no studies regarding the use of IMRT and PT fitting with the inclusion criteria.

The aim of RIT is the achievement of elevated local drug concentration for a protracted time by locally delivering chemotherapy compounds. Moreover, tissue-specific monoclonal antibodies labelled with high-energy β-emitting radionuclides can destroy a large number of tumor cells [67]. Boiardi *et al*. delivered an activity of 5–25 mCi in 26 recurrent GBM following subtotal tumor resection, systemic and locoregional chemotherapy [67]. No severe toxicity was registered and a median PFS of 8 months after the treatment was achieved. Delivering a median activity of 10 mCi in 17 recurrent GBM, Mamelak *et al*. achieved a median OS from the treatment of 6.3 months scoring paresis in 17% of the patients [68].

BNCT is based on the nuclear capture reaction that occurs when nonradioactive boron is irradiated with neutrons of sufficient thermal energy to yield high-energy α particles and lithium nuclei. The effect of α and lithium is limited primarily to boron-containing cells. The modality success is dependant upon a selective uptake of sufficient amounts of boron into cancer cells compared with normal tissues. Preferential uptake of boron into cancerous tissue is achieved using boron carriers [69]. Pellettieri *et al*. delivered 13–27 Gy-equivalent in 12 recurrent GBM following tumor resection [70]. No severe acute toxicity was registered and the study pointed out a median OS and PFS after re-irradiation of 8.7 and 6 months, respectively.

In general, these modalities proved to be feasible and quite safe while clinical outcomes are consistent with the series employing “conventional” re-irradiation modalities. However, considering that enrolled patients often received such techniques at their second or third relapse and that they are often recruited and analyzed along with recurrent anaplastic gliomas, they deserve further investigation as first-line re-treatment in homogeneous patient samples.

## 3. Discussion

The standard of care for patients with recurrent GBM has not yet been clearly defined. Surgery should be considered for all patients even though the benefit of the surgical procedure has to be weighted against the surgical-related morbidity [5]. Because of the extensive brain infiltration, the frequent involvement of eloquent areas, and the risk for further neurological deficits optimal resection is very difficult [6]. In general, only patients with well-accessible tumors and a good performance status are usually managed with this approach [7]. Even though there are series that report a median overall survival from re-operation of 3 to 5.3 months [7,71,72] the best reported outcomes achieve values of about 8 months [8,9,73].

Chemotherapy, employed either alone or in combination with other treatments is probably the most exploited therapy in recurrent GBM. However, the administered agents are not free from toxicity and effective regimens are lacking. Additionally, since the introduction of TMZ, most patients receive CHT during the primary treatment and the bone marrow reserve may be decreased, and patients have poor clinical conditions. To date, re-challenging with TMZ or switching to a non-conventional TMZ regimen has become a common practice that provides six-month progression-free survival (PFS-6) rates of 30 to 48% [10]. Reported median PFS was 4 to 6 months [10]. In addition to TMZ based regimens several non-TMZ based treatment options have been tested in recurrent GBM [74]. A relevant number of drugs and administration schedules were employed. The reported median overall survival for GBM patients was 4.9 to 7.7 months while PFS-6 rates were 20 to 38.4% [74]. More recently, several targeted therapies such as anti-VEGF antibodies, EGFR, PKC/PI3K/AKT and integrin inhibitors have been tested in clinical trials and introduced in clinical practice with very preliminary results [11].

In order to compare the aforementioned results with those achieved with re-irradiation we calculated corresponding outcome values for each re-irradiation technique. Overall, studies that employed 3D-CRT delivered a median dose of 45.5 Gy. Median OS from re-irradiation was 8.5 months. Data on PFS were not reported. Regardless the fractionation, the use of FSRT allowed the delivery of a median dose of 30 Gy. Median OS and PFS from re-irradiation were 8 and 5 months, respectively. The series regarding SRS delivered a median dose of 15 Gy achieving a median OS and PFS from re-irradiation of 10.5 and 4.6 months, respectively. Regardless the type of implant, the use of BT provided a median OS and PFS from re-irradiation of 11 and 5 months, respectively. Considering the relevant differences among studies employing temporary or permanent implants any analysis on the delivered dose can be misleading.

Based on the data we analysed, re-irradiation can represent a valuable salvage treatment option that provides comparable outcomes with respect to re-operation and chemotherapy. Re-irradiation can be accomplished by different techniques and at a first glance they seem equally effective but the results should be interpreted taking into account several issues. Some of them might even represent areas of improvement and/or future research.

### 3.1. Evidence Level and Data Interpretation

There are no prospective randomized trials. Almost all series are retrospective and deal with small to medium patient samples. Merely seven prospective phase I-II studies have been published: none regarding 3D-CRT, two about FSRT, three concerning SRS, and two dealing with BT. Overall, the resulting evidence level [75] is mainly of class III. Moreover, considering that when randomized trials are not available and data mainly come from retrospective studies pooling results is not recommended [76], the meta-analysis methodology could not be applied ultimately precluding a robust analysis of prognostic variables. A solution to accomplish such (meta)analysis could be the application of strictly defined inclusion criteria. Nevertheless, the possibility that errors or biases in individual retrospective studies would be compounded ultimately giving credence to poor quality studies has to be taken into account. As a consequence, also the patient selection in the clinical practice cannot be properly optimized so far. In fact, the inherent variation of tumor and patient characteristics, as well as therapeutic interventions for recurrent GBM patients make comparison of patient groups from different studies unreliable and the results have to be interpreted at the light of several bias. Firstly, BT candidates had tumors without involvement of midline structures, no ventricular disease, and no-deep sited lesions. Moreover, the surgical procedure allowed for maximal safe re-resection ultimately hindering the estimation of the benefit coming from BT. Secondly, SRS series included also patients with potentially adverse prognostic factors not amenable with BT. Moreover, patients with larger recurrent tumors or tumors in eloquent structures were selected to receive FSRT compared with those treated with SRS/BT. As a consequence, potential prognostic variables predicting longer survival were preferentially distributed in favor of SRS/BT. Finally, considering that many patients received additional and different therapies at the time of failure, the end point of survival is a relatively poor measure of treatment efficacy, whereas time to failure after treatment is potentially less subject to the effects of selection bias [77]. Unfortunately, PFS was not available for most series.

Even though only multicenter, prospective trials with well-defined endpoints as well as inclusion and exclusion criteria are necessary to clarify the role of radiotherapy in recurrent GBM some guidelines from existing literature could be useful to best exploit the potential of re-irradiation. The risk of relevant side effects (e.g., radionecrosis) should not be underestimated; hence the patient should be carefully selected. Probably, patients with a good performance status can benefit the most from re-irradiation.

In order to best assess the tumor extension while sparing the surrounding healthy tissues all the information coming from a multi-parametric imaging should be carefully taken into account during the planning.

The tumor size and location can represent useful selection criteria to single out the re-irradiation technique. Large tumors or tumors in eloquent structures, which might be technically ineligible for implantation or SRS, can be safely and effectively treated by FSRT. Such a technique can be delivered both with standard and hypofractionated schedules. Data from literature point out that 36 Gy delivered in 2 Gy per fraction or 30–36 Gy in 5–6 Gy per fraction could represent viable regimens. Patients with small, round shaped, and deep-sited lesions (usually considered not implantable) can be the best application field of SRS. Data from literature support the delivery of 13–15 Gy. Also brachytherapy allows delivering a large dose to the tumor volume while sparing surrounding normal tissue. However, the corresponding invasive procedures involve some surgical risks and require the patient’s hospitalization. The implantation of large tumors (even though feasible) should be avoided. Moreover, it should be offered to patients with no-deep lesions, without involvement of midline structures, and no ventricular disease. Albeit the results obtained with this modality are encouraging regardless the implantation technique, the technical complexity in performing the implants limits its use in current clinical practice. The relevant variability dealing with brachytherapy decreased the possibility to address all the issues dealing with this technique. As a consequence, several topics such as the optimum prescribed dose, dose-rate, isotope, and implant modality have yet to be properly clarified.

### 3.2. Target Definition, Toxicity, and Pattern of Failure in Relation to Imaging

The analysis of the studies revealed that target definition was always based on a morphological approach. By means of magnetic resonance (MR) or computed tomography (CT) imaging almost all authors defined the gross tumor volume (GTV) on the basis of contrast enhancing area following the contrast medium administration. Thereafter, a margin of 2–5 mm was applied to account for microscopic tumor infiltration or setup uncertainties. Rarely, any margin was added to the GTV. On the one hand, this method translated into the aforementioned good clinical outcomes. On the second hand, it provided favorable toxicity rates. Moreover, analyzing the pattern of failure data shows that the vast majority of patients (>80%) continue to recur in the immediate proximity (<2 cm) of the re-irradiated volume. These three remarks apply regardless of the aforementioned variations in target definition as well as the re-irradiation technique. However, conventional CT and MR imaging does not reliably indicate neither the true extent of gliomas or the aggressiveness of different tumor components [78,79]. Therefore, different imaging modalities, such as functional MR imaging, MR spectroscopy and diffusion tensor imaging (DTI) as well as PET scans have been used to visualize the clinically relevant volumes. Early studies have shown the feasibility of incorporating functional and spectroscopic MR images [80] as well as DTI [81] into treatment planning. At the same time, a significant change in target location, volume and shape compared to conventional MR imaging has been demonstrated. By means of amino acid PET, Grosu *et al*. pointed out similar conclusions highlighting a very high sensitivity and accuracy of such modality [82]. From this standpoint, the better assessment of tumor extent could improve the tumor irradiation and ultimately the clinical outcomes as well. Grosu *et al*. recently demonstrated that treatment planning based on both biological (amino acid PET/single photon emission CT) and conventional imaging (CT/MR) was associated with improved survival in comparison to treatment planning using CT/MR imaging alone [83]. However, the increased complexity of target definition could provide more difficulties to comply with dose constraints and could increase the risk of toxicity if larger amount of healthy tissues were included into the target. From this standpoint, it may happen that one technique takes advantage over the others with respect to a specific clinical scenario, ultimately allowing the assessment of the best application setting for each technique. Such an issue represents at the same time an area of improvement for the current clinical practice and an argument worthy of further investigations.

### 3.3. Association with Chemotherapy

The EORTC/NCIC randomized trial [3] has shown unequivocally that addition of TMZ to RT provides both progression-free and overall survival advantage with respect to RT alone in GBM patients. Before the introduction of TMZ the addition of CHT to RT had been a controversial issue in this clinical setting even though a meta-analysis pointed out a survival benefit from this strategy [84]. Hence, the radio-chemotherapy association could represent the best approach in the attempt to improve the clinical outcomes at the recurrence. However, the combined modality has to face some drawbacks. Firstly, administered agents are not free from toxicity. Hence, considering that patients harboring recurrent GBM often have poor clinical conditions their administration could be troublesome. Secondly, since the introduction of TMZ most patients receive CHT during the primary treatment so that at recurrence the bone marrow reserve may be decreased ultimately affecting negatively the patient compliance. Moreover, the combined modality seems more prone to increase the side effects so that physicians are reluctant to employ such strategy. Finally, re-irradiation techniques such as BT and SRS feature delivery procedures that hinder “per se” the concomitant administration of CHT.

To date, only few studies reported results concerning concomitant chemo-irradiation of recurrent GBM. Some series employed “old generation” agents such as paclitaxel and topotecan [24,29] while others re-challenged the current standard association RT-TMZ [31,37]. Only one study tested a “new” drug, such as Bevacizumab [34]. Such lack of data does not allow drawing reliable conclusions concerning the efficacy of this strategy and points out weak evidence regarding its feasibility and safety. However, several targeted therapies such as anti-VEGF receptors antibodies, EGFR, PKC/PI3K/AKT and integrin inhibitors recently demonstrated their safety even when administered together with RT-TMZ [11]. At the light of their safe profiles as well as the aforementioned potential benefit, radiation oncologists should consider the CHT-RT association as one of the most promising areas of research in the attempt to improve clinical results in recurrent GBM. From this standpoint, considering their multi-session delivery, conventional or hypofractionated regimens best fit with this goal.

## 4. Conclusions

Despite of the low evidence level, re-irradiation of recurrent GBM employing high precision techniques provides survival prolongation and delays disease progression with acceptable toxicity rates. However, it is not a curative treatment and it is limited to selected subgroups of patients. Therefore, a further therapeutic improvement is needed. Such amelioration can be achieved through well-designed prospective trials that address both issues concerning the optimum application field for each technique (such as the optimal prescribed dose and the volume-cutoff of the target) and new investigational areas (such as the implementation of new imaging modalities into the treatment planning, as well as the radiochemotherapy association).
